# Interprofessional collaboration to support patients with social problems in general practice—a qualitative focus group study

**DOI:** 10.1186/s12875-022-01782-z

**Published:** 2022-07-04

**Authors:** Thomas Kloppe, Britta Tetzlaff, Claudia Mews, Thomas Zimmermann, Martin Scherer

**Affiliations:** Department of General Practice and Primary Care, University Medical Centre Hamburg-Eppendorf, Martinistraße 52, 20246 Hamburg, Germany

**Keywords:** General practitioners, Social care system, Social care practitioners, Social professionals, Social determinants of health, Cooperation, Collaboration, Interprofessional, Qualitative study

## Abstract

**Background:**

Social problems of patients such as family or work-related conflicts as well as financial difficulties affect the individual health situation and the treatment of diseases in general practice. General practitioners (GPs) would like to have direct access to professionals in social care services. In Germany, there are many different social care facilities for people with a wide range of social problems. As the social and health care systems hardly interact collaborations between social professionals (SPs) and GPs are rare exceptions.

This study explored perspectives of GPs regarding their patients with social problems in combination with the perspectives of SPs. Aim of this study was to explore how a systematic interprofessional collaboration between GPs and SPs could be realised.

**Methods:**

We carried out a participatory sequential qualitative study design consisting of two focus groups with GPs, two with SPs and two mixed-professional focus groups with GPs and SPs. The focus groups were conducted with semi-structured moderating guidelines and analysed with a qualitative content analysis approach using inductive and deductive categories.

**Results:**

GPs view themselves as the first point of contact for their patients' social problems. For persistent social problems, they expressed a desire for support and SPs were willing to provide this. We developed a stepped care implementation model for a systematic cooperation consisting of nine collaboration strategies. These strategies included: index or website of social care services, referrals to the social care system, using flyers and posters of social care services, direct contact/hotline to local social care services, participation in meetings of social care facilities, involving physician assistants, external social care advice service in GP rooms, implementation in education and training and access to volunteers.

**Conclusions:**

Our stepped care implementation model for a systematic cooperation of GPs and SPs could be a feasible need- and resource-oriented approach for the collaborative care of patients with social problems to improve their medical treatment in most western healthcare systems. GPs and SPs are ready to generate the necessary evidence for policy makers in high quality RCTs.

## Background

As socio-economic factors, social problems such as unemployment and homelessness, financial difficulties, or private circumstances such as partnership problems, family and work-related conflicts affect the participation and functioning of people [1]. Social problems, as categorised in the Z-chapters of ICD-11 [2] and ICPC-3 [3], are social determinants of health [4] and affect health at the individual level with a significant impact on the course and the treatment of diseases. People with social problems are more likely to suffer from mental impairment, cardiovascular diseases, multimorbidity, have a shorter life expectancy and a poorer quality of life [5]. At the same time, multimorbidity or severe illnesses such as cancer or chronic inflammatory bowel disease have a considerable influence on the development of social problems likes unemployment, financial difficulties, and interpersonal conflicts [6, 7]. As a result, new medical problems or deteriorations may occur [8].

In a previous study [9] of the epidemiology of social problems in primary care, we were able to show that out of 1,844 GPs in northern Germany, 47.8% reported that they encountered at least five social problems every week: mainly financial difficulties, problems with work or unemployment, loneliness, problems with relationships and cultural problems e.g. related to discrimination or integration [9]. Bikson et al. [10] found that patients in the US reported similar social problems related to finances, personal stress, transportation, (un-)employment and legal issues. Approximately one third of the surveyed primary care patients with social problems (*n* = 684) stated that they would like to see a social worker.

Many socially disadvantaged persons first seek support from their GP [11–14]. Zantinge et al. [15] showed in a survey of 226,920 participants that patients with psychological or social problems had almost twice as many GP contacts as patients with somatic problems only. The GP can be a viable point of contact for patients with social problems [16, 17]. Often, those affected appreciate the non-stigmatising, confidential and discrete character of general practice [18]. It is not visible to the outside world that they have a social problem when they consult their GP [19]. Most GPs try to find pragmatic and appropriate solutions with their patients. For further support, GPs would like to have a contact person within a social facility [9, 18, 20].

A study by McGregor et al. [21] suggested that social work in primary care that addresses social problems also contributes to more positive health outcomes, such as improved subjective health, functioning and self-management, lower psychosocial morbidity and reduced barriers to treatment and health maintenance. Many examples of these collaborations are implemented in the United Kingdom [22], in Ireland [23], in Canada [24] or New Zealand [25].

However, in Germany, there is no interprofessional collaboration between general practice and social care [21], as the healthcare system and the social system hardly interact. Therefore, it is very challenging for GPs to find an appropriate social care facility [20]. In consequence social problems are not addressed until they become a severe symptom of a disease. Specific guidelines or well-established approaches like social prescribing [26] are almost non-existent in Germany [27] and the knowledge of social care services and their methods are generally poor among GPs [28]. There are only a few pilot projects directed at social problems in primary care, such as the “Patient-oriented primary and long-term care centres (PORT centres)” [29] or the “Community-based Health Service provider (Gesundheitskiosk)” [30]. The funding or continuation of these projects remains uncertain and full-scale implementation in the next few years is unlikely. At the moment, GP’s individual approaches to dealing with patients with social problems often depend on their own time resources, personal commitment and attitude [31]. We suspect that such unsystematic detection and treatment of social problems in general practice blocks valuable time, financial resources and leads to overutilisation of ambulatory medical care in Germany and elsewhere [32]. Interprofessional work as a complex concept defined by the relationships and interactions between different health and social professions [33] is considered as a strategy that can optimize health-services, strengthen health systems, and improve health outcomes [34].With the objective of promoting the development of interprofessional collaboration to support patients with social problems in general practice in Germany, our study aims to address three key questions:

1. which social problems occur most in GP consultations for which types of patients,
2. which social care facilities could offer helpful services for the social problems of the identified patients, and
3. what kind of collaboration between the social care sector and GPs might support patients with social problems?

## Methods

### Study design

We conducted an explorative qualitative study with a total of six focus groups (two with GPs, two with SPs and two mixed-professional focus groups with GPs and SPs) in a participatory sequential study design. We choose focus groups because group dynamics should provide rich data with a high diversity of opinions and innovative ideas [35, 36].

### Recruitment

The study took place in Hamburg, a large city with 1.8 million inhabitants in the north of Germany. Participants were selected following a purposive sampling approach [37]. Focus group participants were recruited in two steps:

1. Five hundred GPs were randomly selected and invited to the study by mail, using the register of the Association of Statutory Health Insurance Physicians. Based on several socioeconomic indicators, we chose GPs from areas with a "very low" to a "medium" status level because of higher likelihood of social problems [38].
2. Based on results of the GP focus groups, we invited matching facilities within the social care system. For example, one GP focus group discussed patients with financial difficulties, so we invited a debt advisory service. In Hamburg, there are several different help and treatment options for people with social problems. Social care facilities with a public funding are listed on the website of the city of Hamburg. We recruited facilities in different city districts to gather a perspective that is as broad as possible. In addition, we contacted further facilities that were specifically mentioned in the GP focus groups. Overall, we invited 25 facilities to participate in focus groups. For the SP focus groups, we invited social professionals who work directly with clients. For the mixed-professional focus groups, we mainly invited managers and policy makers.

Inclusion criteria for GPs were: (a) specialists in general practice or internal medicine, (b) provision of regular general medicine (including home visits, no focus on homeopathy, naturopathy, or psychotherapy), (c) treatment of at least 500 patients per quarter, (d) located in Hamburg, (e) informed consent. A total of 31 eligible GPs showed interest to participate. Inclusion criteria for social professionals (SP) were: (a) state certified social workers with (b) job experience in a social care facility, (c) competence about social problems described by the GPs for which support is needed and (d) informed consent.

### Participants

We included 16 GPs as per the characteristics of social status of the local area, professional experience, and sex, and 19 SPs. Six of the 25 requested facilities were unable to participate due to time constraints and illness. Each participant was contacted by the researchers (BT/TK) to review inclusion criteria, explain the specifics of the study, obtain informed consent and to schedule participation in the focus group. Characteristics of study participants are shown in Table 1.

**Table 1:** Characteristics of participants (*N* = 35)

		**General Practitioners**	**Social Professionals**
**Total**		***n* = 16**	***n* = 19**
**Sex**	Female	11	14
	Male	5	5
**Age in years**	30 – 39	1	4
	40 – 49	3	5
	50 – 59	8	6
	60 – 69	4	3
	Not specified		1
**Total professional experience in years**	< 10	2	2
	10 – 19	4	7
	20 – 29	6	3
	30 – 39	4	4
	> 40		2
	Not specified		1
**Specialist title**	General practitioner	14	
	Internist	2	
	Social worker		13
	Nursing consultants		1
	Management and cross-institutional stakeholders		5
**Practice organisation**	Single practice	5	
	Group practice	10	
	Not specified	1	
	Voluntary sector		9
	Statutory sector		10
**Social care facilities**	**SP focus group 1 (*n* = 7)**		
	Drug and alcohol advice centre		1
	Senior’s counselling centre		2
	Psychosocial counselling centre		1
	Debt advisory service		1
	Youth service centre		2
	**SP focus group 2 (*n* = 6)**		
	Family centre		1
	Psychosocial counselling centre		1
	Senior’s counselling centre		1
	Self-help group network		1
	Youth job agency		1
	Local authority children’s services		6
	**Mixed-professional focus group 1 (*n* = 2)**		
	Debt advisory service		1
	Family centre		1
	**Mixed-professional focus group 2 (*n* = 4)**		
	Welfare association		1
	Local authority for health promotion		2
	Local authority for community services		1

### Ethics statement

This study received ethical approval from the Ethics Committee of the Medical Association of Hamburg on 8 February 2020, reference number LPEK-0107.

### Setting/procedure

Our study follows a three-staged explorative sequential design:

1. Two focus groups with ten GPs were conducted and evaluated.
2. The results were presented in two successively held focus groups with a total of 13 SPs.
3. The mixed-professional focus groups with six GPs and six SPs were conducted with the aim of complementing and agreeing on the results of the previous focus groups.

We conducted all six focus groups with a total of 35 participants between May and August 2020 via video conference, lasting for two hours each. Moderation was performed by BT, a female trained occupational therapist and post doctorate researcher with experience in moderating workshops and focus groups, with support from TK as co-moderator. He is a male trained state-approved social worker and post doctorate researcher. A student assistant recorded focus groups for verbatim transcription. The moderator followed a semi-structured guide; topics are shown in Table 2.

**Table 2:** Semi-structured moderating guideline

**GP focus groups**
• What social problem have you discussed most often in your practice? • How do you talk to your patients about these and other social problems? • In what cases have you already recommended or referred patients to social care professionals? • What type of collaboration should be established with social care professionals to address patients' social problems in general practice?
**SP focus groups**
• How could your facility address specific social problems of GP patients? • What could a structural cooperation with GPs look like?
**Mixed-professional focus groups**
• Do you have any other ideas for addressing social problems in general practice that did not emerge in the previous focus groups? • Based on your capabilities and patient acceptance, how would you rate the feasibility of each idea?

### Analysis

BT reviewed all transcripts, which were produced and pseudonymised by the student assistant. The qualitative analysis was based on a mixed deductive and inductive development of categories. BT and TK analysed the data together, providing a high degree of intersubjective verifiability in the interpretation of the data [39]. The software MAXQDA 12 [40] was used to structure the qualitative data analysis.

We used content analysis across all six focus groups to answer the research questions subsequently. The main categories of the focus groups were 1) Social problems of patients in general practice, 2) General practitioners’ course of action, 3) Experiences with collaboration 4) Ideas for future cooperation and 5) Barriers and facilitators for the implementation. We ranked these ideas according to the needs of patients and required resources.

Patient types and their social problems were developed collaboratively during the GP focus groups. For this purpose, we documented and clustered all statements during the focus groups and consented the results at the end of each focus group. In addition, we identified specific social care services which we invited to the SP focus groups.

## Results

### Social problems of patients in general practice

The social problems of patients, reported by GPs (*n* = 10) in the first two GP focus groups, were categorised into six specific patient types with typical social problems, shown in Table 3.

**Table 3:** Patient Types and social problems, reported by GPs

Young adults (16–26 years)	Single mothers, mothers with many children	Adults in complex problem situations	Older patients	Entrepreneurs (e.g., fruit farmers)	Patients with drug-related harms
– Self-worth problem, – Puberty, – Lack of perspective, – Absence from school or training place	– Childcare and parenting issues, – Separation/ divorce, – Too small apartment, – Financial problems, – Violence in the family	– Loss of jobs, – Mobbing at the workplace, – Re-integration into the job, – Fear of financial difficulties/poverty, – Loss of apartment, – Care of children and elderly people, – Domestic violence, – Divorce	– Loneliness, – Lack of education, – Lack of independence – Poverty, – Depression in old age, – Moving into a nursing home	– Bankruptcy, – Threatened self-employment, – Fear of financial difficulties, – Domestic violence	– Social psychiatric problems, – Homelessness, – Workplace issues, – Poverty

Across patient types and social problems, GPs reported additional barriers like various foreign languages, cultural differences in the perspective of the disease and a lack of knowledge about social care services.

*"[...], from care services to basic minimum income, applying for a degree of disability, all these formalities, many people don't take this up because they don't know the possibilities [...]. (GP-FG1, GP2, paragraph 12)*

### General practitioners’ course of action

GPs defined themselves as the first point of contact for their patients’ social problems.

*"Patients often come to their GP for the first time with a problem (.), which means it's not that I try to mediate immediately, but […] in the first contact to listen and say "ah ok, there could be a problem" and perhaps to discuss it further at the next appointment […].” (GP-FG1, GP3, paragraph 78)*

They assumed that they take care of social problems a long time before social care facilities are involved, because they are often the first address when patients are unable to work, need medical care or severe events occur. The GPs described that they have a long-standing relationship with their patients, are familiar with their medical history and usually also know about their social environment. They defined the joint treatment of somatic symptoms and psychosocial problems as core principle of primary care. GPs reported that they apply simple conversation techniques to address problematic situations.

*"Patients come to the doctor because they have stomach pains and not because they have lost their job (...) usually, [...] to recognise this is already [...] a challenge in part and making patients understand that their stomach pains are perhaps related to their job loss [...] and then establishing confidence and saying "okay, I am a doctor, I can say that stomach pains are sometimes related to a job loss" [...] and then you can work on the social problems." (GP-FG1, GP3, paragraph 80)*

To counsel patients with social problems without time pressure, GPs schedule several appointments in the off-peak hours.

”*And then maybe giving the patient a new appointment where it is not so busy, where we have [...] the chance to address this topic specifically, without the time pressure that you might have [...] in addition to taking blood and so on". (GP-FG1, GP2, paragraph 88)*

As a second possible way of addressing social problems, some GPs described that they recommend a local social service to their patients.

*"[...] Then I tried to connect him, so at our place there is [a local social care facility] where you can make appointments with a low barrier and talk to social workers who are supposed to deal with exactly this type of problem [...]" (GP-FG2, GP5, paragraph 77).*

### Experiences with collaboration

However, only half of the GPs reported that they have been in contact with social care facilities. These GPs described contacts with staff from women’s shelters, children’s services department, and nursing services.

*"[…] we also have frequent contact with staff from the women's shelter, […]. About two or three times a month.” (GP-FG2, GP2, paragraph 96)*

Regular cooperation with social care facilities only took place in special substitution programmes for patients with drug-related harms.

*"These [psychosocial counselling] facilities are part of the substitution concept, and the patient has to sign, if he wants to be substituted, that the doctors are allowed to have a complete exchange with the social workers, we don't have that in any other area.” (GP-FG1, GP5, paragraph 109)*

In addition, they particularly emphasised that contact with special caregivers is valuable for non-German speaking patients.

*"[...] what I like a lot is that recently I have more foreign patients who have only limited knowledge of the German language or who are not very familiar with the health care system, that a special person has been set up who comes into the doctor's consultation.” (GP-FG1, GP2, paragraph 105)*

GPs described a lack of knowledge about suitable social care facilities and services. They did not feel familiar with the quantity of available social care services, although when they had acquired some knowledge during their professional experience.

*"[…] the system of social services is extremely broad, therefore I often lose the overview." (GP-FG1, GP1, paragraph 68)*

The participating SPs had a strong interest in networking and cooperation for an early and more effective allocation of people with social problems. They would like to provide individual advice to physicians and conduct briefings to increase awareness of social care services. However, in their experience, GPs rarely attend information events or local network meetings.

They also assume that the lack of knowledge and the exclusive consultation of individual social problems by physicians could hinder comprehensive consultation. To deal with the complexity of social problems, comprehensive counselling should take place at the beginning of the process to ensure that several issues can be addressed. Otherwise, patients could be frustrated by inadequate services.

*"Uh, as I said, we have noticed that, if he [a GP] takes brochures from three or four providers out of the drawer and gives them to people, the way to comprehensive counselling is actually cut off immediately.” (SP-FG2, SP4, paragraph 72)*

Representatives of the social care services described a lack of structures as the main barrier for professional cooperation. To foster collaboration, financing must be provided for both sides, because regular exchange requires additional work.

*"I think that things like that would also have to be supported financially. I don't think any GP goes there if he doesn't get money for it and it's the same with the social facilities. We have a great interest in networking but that's always just on top, […] and up to now it hasn't been supported and I think it would be important that a certain amount of working time is available for it." (SP-FG1, SP3, paragraph 38)*

### Ideas for future cooperation

Each focus group developed different strategies to facilitate systematic collaboration (Table 4).

**Table 4:** Strategies for a systematic cooperation of GPs and SPs

**GP focus groups**
• Index or website of social care services • Referrals to the social care system • Direct contact/hotline to local social care services
**SP focus groups**
• Participation in meetings of social care facilities • External social care advice service in GP rooms • Implementation in education and training
**Mixed-professional focus groups**
• Using flyers and posters of social care services • Involving physician assistants • Access to volunteers

### Stepped implementation strategies for a systematic cooperation of GPs and SPs

**Fig. 1:**
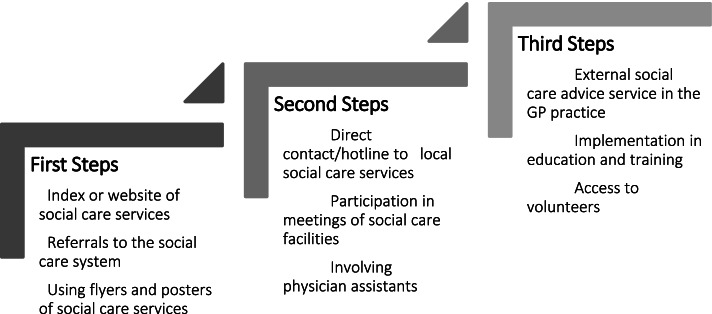
Stepped implementation model for a systematic cooperation of GPs and SPs

For a feasible need- and resource-oriented implementation, we developed a model with stepped priorities for the resulting nine collaboration strategies (Fig. 1). These emerging strategies are explained in more detail below.

#### Index or website of social care services

From the GPs’ perspective, a detailed and sorted list of general social care services should be available. The central file location could be the website of the responsible General Practitioners’ Association.

*"[...] and sometimes I would wish [...] that for example on the website of the General Practitioners’ Association [...] that there is support: where can I get a contact for a specific problem? Or what could I suggest?" (GP-FG1, GP2, paragraph 84).*

SPs mentioned that lists are not very promising because they are usually exclusively useful for the person who creates them, because only they know when they should be used and how.

*"Lists make sense only for the people who create them […] the gain in knowledge is highest there. […]". (SP-FG1, SP2, paragraph 116)*

In addition, lists and website become outdated very quickly if they are not permanently maintained. In the mixed-professional focus groups, the idea of a list was expanded to a digital map showing different social care facilities with defined contacts in each area. One of the participants stated that a local list that GPs would create for themselves would be more useful. However, a list or map would not replace personal contact.

*"As the previous speakers have already said, this is no substitute for personal contact. I think that referring people, referring them with a good feeling, also requires a kind of familiarity with the person or that I have already heard the structure mentioned by someone else, that this is good, that this is a good offer, so that’s something else that is also needed, but I could also imagine a digital list that might be helpful for finding the way through this whole jungle of extremely differentiated services." (MP-FG2, SP6, paragraph 52)*

GPs also wanted to talk to someone directly if needed. For this purpose, they wanted to know the names of the SPs, what they could do for their patients, and whether they felt comfortable recommending patients to them. For a list or website to be successful, care must be under the professional direction of an association, university, or local government.

#### Referrals to the social care system

The participating GPs mentioned that a direct “referral to a social care service” in form of a formalised medical referral to another specialist could be a strategy of cooperation. To protect the privacy and personality of their patients, they did not want to receive feedback from social care facilities.

*“I provide information and then I ask the patient next time, and if the patient [...] decides against it or says, oh no, somehow, I'd rather keep drinking or keep being sad or something, that' s his right too. Then you may not have the feeling that you have received information [by a SP] that he [the patient] did not want to tell you himself. So maybe, maybe this feedback is a bit too much. So maybe you have to be careful." (GP-FG1, GP3, paragraph 131)*

The SPs generally supported this idea, as it could enhance the value of social care services, reduce barriers, and increase commitment.

*"[…] and I think the idea with the referral form […] is good because it upgrades the whole consultative process." (SP-FG1, SP3, paragraph 38)*

On the other hand, such a formalised referral could reinforce an exaggerated sense of entitlement of patients to quick solutions and jeopardise the principle of free choice.

*“[…] the basis for self-help is that people can call us because they have to be able to participate in meetings regularly on their own. Yes, that is the basis of motivation and activation, but not care and referral.” (SP-FG2, SP3, paragraph 60)*

Based on this, no added value was seen in the mixed-professional focus groups for the implementation of formalised referrals, apart from the mentioned “commitment” of social care services. Non-formalised recommendations based on a good knowledge of the social care system remained very welcome by GPs and SPs.

#### Using flyers and posters of social care services

One GP suggested an improved use of flyers and posters in the waiting room. Newly designed flyers, or posters should include a brief general characterisation of general social care services. In addition, these materials should encourage patients to contact the GP about their social problems. This approach could reduce barriers for patients without overburdening the system if GPs are able to refer to social services quickly. These flyers and posters could also be displayed in pharmacies, on public transportation, or in other waiting areas to make people aware of the possibility of talking to their GP so they can get in touch with social care services.

Some participants also did not believe that patients would use impersonal flyers to contact GPs. They emphasised that GPs should meet social facilities first to make recommendations. Overall, flyers should point out the benefits of social care services rather than specific problems and their consequences.

*"[...] first an informational event, after that, when we stand behind these flyers, and we are informed, after that, I would display it that way and, in my eyes, these are not only "problem flyers" they could be called "what Hamburg is doing for you" [...]." MP-FG2, GP 8, paragraph 58)*

#### Direct contact/hotline to local social care services

Another idea of collaboration in addressing patients' social problems in the GP practice are local contacts to social care facilities that provide universal support for a wide range of problems and are easily accessible at low thresholds. GPs argued that these “neighbourhood social care services” (GP-FG1, GP4, paragraph 149) should be staffed by SPs who are familiar with the various options of the welfare state, local public services, and other social facilities of the local infrastructure. Specific counselling services could be offered for clusters of social problems and types of patients at different times, e.g., for 16 to 26-year-olds or for senior citizens. All services should be multilingual to enable counselling for international patients. An additional integrated hotline offering brief low-threshold counselling was also considered helpful.

*“I would like to see a 24-hour hotline, […] where you can at least make the first contact in a concrete way, […] “so here's the phone number, there is someone who will listen and try to help you […].” (GP-FG2, GP2, paragraph 110)*

SPs would welcome general advice from local social care service especially for GPs. SPs could recommend appropriate services to GPs, and GPs could provide comprehensive advice to their patients without having to spend a lot of time and effort on specific enquiries. At least two consultants would need to work in such an office to avoid long waiting times.

In the mixed-professional focus groups, the strategy of a local, low-threshold social care services with a hotline has been further elaborated. There, SPs could provide information-seeking people with information about problem-specific local social services.

*"[...] a kind of telephone office that first gives information about the nearest agencies in the area and then a kind of 360-degree advice about who is best responsible for this or that in the neighbourhood [...]." (MP-FG2, SP5, paragraph 22)*

An indispensable basis for the consultants must be the first mentioned index or website of social care services.

*“[…] of who is actually available for what". (MP-FG2, SP6, paragraph 40)*

#### Participation in meetings of social care facilities

SPs pointed out, that a personal advice for GPs could be integrated in regular district conferences of social facilities or into newly established “neighbourhood meetings”. These meetings could provide general information about nearby local social care facilities and would allow a vivid discussion about anonymous cases at a “round table”. To date, there have been different attempts for those meetings without any physicians participating. Participating SPs considered that aspects of GP quality management, the awarding of continuing education points could motivate GPs to participate. If these meetings took place as online, GPs would be able to join in briefly to find case-related solutions.

*“Videoconferences are definitely a time-saving factor, maybe that is more feasible for GPs, to call in for an hour somewhere and a counselling facility introduces itself or introduces the counselling system in the district. Maybe that is more feasible than getting all the GPs at the same table [...].” (SP-FG1, SP6, paragraph 48).*

The GPs in the mixed-professional focus groups showed interest in participating in regular local network meetings. They considered an annual meeting for information purposes only to be sufficient. For meetings in which specific problems cases are discussed, an interval of 6 weeks was suggested. In such a meeting, ideally all local social facilities would be present to enable personal contact. Of course, it would be ideal if the necessary time resources were available in the medical and social system.

*“Exactly, so um, I think that this would have to be done systematically. I don't know what it's like for doctors, but I think it's always on top, and we've actually had interdisciplinary case discussions, but that's really a tour de force to keep it up, […] and if you as a general practitioner also have the capacity to do that.” (SP-FG5, SP3, paragraph 44)*

An additional suggestion was to link these meetings to existing Medical Quality Circles, District Doctors’ Meetings of the Association of Statutory Health Insurance Physicians or obligatory events within formalised Disease Management Programmes in Germany could be possible platforms to involve SPs from various social care facilities.

#### Involving physician assistants

The strategy of rethinking the role of physician assistants in counselling patients with social problems emerged from the SPs in the mixed-professional focus groups. Physician assistants could maintain contacts with social services and motivate patients to seek these services. It might be an advantage that physician assistants are closer to patients’ social needs and backgrounds than academically trained GPs.

*"[...] but often we have also made the experience that there is not necessarily much time in the exchange with the doctors, or that many things have already led to a rejection by the physician assistants, that homeless people have not crossed the threshold or that, there is a lot of catching up to do and maybe another ear is another ear, so that this source could perhaps be expanded again as, um, as someone who also refers or with, um, yes, again seeks the conversation differently, perhaps, [...].” (MP-FG2, SP6, paragraph 40)*

GPs had concerns about the time resources of physician assistants and about data protection during a consultation at the counter in the GP practice. However, it might be an option to train physician assistants who could provide advice, for example to older patients during a home visit.

*"[...] we have a physician assistant who has participated in a further training course as a non-medical practice assistant and who can also carry out home visits independently, [...] and, therefore, in close consultation with us, she informs the patients or gives us important feedback after home visits, if there is a need for further action. It is of course, mainly the elderly patients who need further help, but that is for sure, would certainly be a practical way of doing so via the doctor's practice, via a physician assistant there, which is trained and specialised accordingly.“ (MP-FG2, GP7, paragraph 56)*

#### External social care advice service in GP rooms

Another widely discussed strategy was the provision of regular external social counselling hours directly in interested GP practices.

*"If a general practitioner is interested, it would be a good idea to discuss (.) whether or not to have a kind of counselling through the counselling centre in the practice.” (SP-FG2, SP5, paragraph 98)*

SPs and GPs emphasise that the option of social care advice in the familiar surroundings of the GP practice could be a minor hurdle to seeking and accepting social support. SPs could move some of their open consultation hours into the GP practice. Only the appointments would need to be organised and bundled by the practice and a room would be needed. In the mixed-professional focus groups, this approach was expanded to include the idea of offering joint interprofessional counselling in the GP practice.

*"[...] I think it is even better to say that we invite [...] directly to the consultation, so if a certain family XY is present, then we can do the consultation together". (MP-FG2, GP3, paragraph 68)*

Even specialised counselling facilities, for long-term care or the care of patients with drug-related harms could offer consultation hours in the GP's practice. For these specialised consultations, several GP practices could join forces and refer eligible patients to the GP in charge.

*"In the practice is a great idea. […], but with so many specialties, I don't think there are enough patients left for a consultation of about three hours on Tuesday. You'd have to team up with several doctors, […], because somehow, if there’s a social professional sitting there and no one comes, it's no fun.” (MP-FG2, GP3, paragraph 68)*

At the end, SPs expressed some concerns of limited capacity. Often specialised social care services are overburdened, and pressure with refinancing is quite common.

#### Implementation in education and training

A lack of awareness of local social care services could be counteracted by addressing social problems in medical education and training of doctors. Regular accredited exchange and information events, which take place as time-saving online formats, should be a part of regular quality management and continuous education in GP practices.

*"Doctors must continue their training. One could knit a system that would include, a biennial training program ‘What are the social facilities in the neighbourhood?’". (SP-FG1, SP6, paragraph 48)*

GPs liked the idea of addressing social problems in medical education. Additionally, online training courses or lectures could be offered as part of the established continuous GP training. The most important factor for GP is the implementation into the specialist training. SPs emphasises a strong willingness to support the education and training of GPs.

#### Access to voluntary work

The increased use of volunteer networks was briefly discussed. These services are usually initiated and maintained by professionals in social care facilities. They connect volunteers with persons in need of assistance. GPs reported that many of their patients have minor needs and that volunteer platforms would be a practical, low-threshold tool for support. For example, to counteract an elderly lady's loneliness, access to a volunteer network could be used to arrange direct contact.

*"[...] and then I would like to see if there is a volunteer’s exchange where we can access that. [...] where a doctor or other staff member can pick someone out of the system and have a direct, a direct impact. We have the ideas and then it ebbs away in part because you don't always have access to the person in question. [...]" (MP-FG2, GP8, paragraph 32)*

### Barriers and facilitators for the implementation

For the implementation of the presented collaboration strategies for more systematic collaboration between medical and social care services, several barriers were identified. Participants emphasised the need for more general consultations hours to be available in social care facilities to advise patients. Some of these facilities had to close due to funding shortages. An extended use of social care services as well as the use of flyers and advertising posters would cause additional costs. A further step should include the mandatory involvement of public payers like health insurance funds, long-term care insurance funds and other local institutions.

*"[...] in the next step, you have to interest funding authorities, no matter who they are at the end, but of course health insurances, care insurances and of course also the city are involved." (MP-FG2, SP2, paragraph 76)*

One lever for cooperation between GPs and SPs within the urban area is the integration of health issues into planning local social infrastructure by the relevant government authorities. In the city of Hamburg, where we conducted the interviews, some planned structures could be considered as beneficial factors for better cooperation. The first local community health centres are planned, and various funds intended for the social development of a district could be used for the implementation of the developed ideas.

*“Next year there will be some experimental integrated health care centres in each district, maybe that is a good idea [...] for the authorities to promote such structures, to link social care and health care per district and, most important, to establish this network well, maybe that would be a place where it would be possible to dock this contact person, this ‘gatekeeper’ model from the medical area for the psychosocial help area [...]." (MP-FG2, SP6, paragraph 40).*

## Discussion

In our study, GPs pictured themselves as a first point of contact when it came to handling social problems with their patients and they want to offer optimal counselling in their practice. These confidential consultations, in which social problems are explored in depth, are a well-known source of the particularly trusting relationship between GP and their patient [41–44]. For complex, persistent, or urgent social problems [45–47], GPs expressed a desire for support and it has become apparent that SPs are willing to provide this support [28].

Based on these assumptions, we developed a stepped implementation model for a systematic collaboration between GPs and SPs to be the core component of a complex intervention. But not all these steps are equally relevant, effective, and efficient. Overall, the most effective and the most challenging approach seems to be the integration of an additional social care advice in German GP practices [20, 48]. This strategy involves social workers from a social care facility providing regular counselling consultations directly in the GP's practice. This ensures consistency in the services offered, direct contact between the professions, a low-threshold service for patients, and no losses due to patients having to move to a different location. This approach promises exceptionally good prospects of being successful, as it has been widely tested in other countries [49] and in heterogeneous settings for at least 80 years [50–52]. This concept has shown promising effects on subjective health, self-management of chronic conditions and the reduction of psychosocial morbidity, emotional role functioning, medication safety and further barriers to medical treatment [21, 53, 54]. The non-stigmatising, confidential and anonymous character of a general practice empowers patients to seek earlier support [19]. This is supported by the IRIS study from Feder et al. [55]. They show that involving the whole practice team, integrating reminders into the practice software and a formalised referral to the appropriate social care service is a very effective way to support patients affected of domestic violence [55].

However, the direct integration of social work into general practice will take many years in Germany, because GPs mostly work in small single practices and the health care system is strictly separated from the social care system [56]. Meanwhile, more pragmatic methods are needed for easier referrals to the social care system [20, 28]. Using flyers and posters of social care services in waiting rooms and in consultations are a simple first step in making patients aware of general and specific social care services [19]. For individualised support and referrals, GPs need easy access to an up to date index on a website [57]. The minimum scope should include general social and specialised counselling services focusing on financial or housing problems [18].

Further support for treating a wide range of diseases, anxieties and health-related behaviours of individuals and population groups need a more ambitious social prescribing approach which involves “link workers” [26, 49, 58–60]. In our study, GPs wished for a hotline or direct telephone contact to a SP in a social care facility to clarify specific questions directly and quickly. The realisation of this service would not require a completely new infrastructure, [61]. The participating SPs showed up ready to offer an effective short telephone consultation for GPs as part of their regular work.

Ideally, a “link worker” in general practice has a high level of experience, comprehensive training programs, frequent communication with the GP and a regular supervision to ensure reliable support [62, 63]. Nevertheless, these essential supportive contacts with SPs take up valuable time which is not remunerated additionally. A pragmatic approach for SPs and GPs might be occasional participating in existing local meetings of social care facilities, which are well established in Germany. Even the involvement of physician assistants for maintaining contacts could be a promising approach [20, 64].

The “link worker” could integrate the diverse volunteer’s sector as well. Volunteers could support patients affected by social problems with small services, relieving conversations, and mentoring [65, 66], even in situations where social care facilities are overloaded [67]. For this purpose, it is outstanding that social work must ensure a tailored assessment and a clear support package to patients and GPs.

One of the biggest challenges is developing a shared culture [68] between SPs and GPs, which have had little interaction in the past [25]. GPs described that a clear understanding of social work capabilities and limitations is essential to avoid inappropriate referrals [63]. But after reviewing 35 qualitative studies, O'Carroll, McSwiggan and Campbell [69] found a lack of knowledge in this field. An emerging interest in interprofessional work mainly arises when physicians had contacts with the social care sector during their education or training [69]. Consequently, medical schools and training facilities should create opportunities for interactions with social care facilities to establish a common understanding. The integration of social determinants of health and social care services into medical education and GP-training, which was emphasised in our focus groups, is rare in Germany [70] and elsewhere [69]. Curricula are usually rigid, but every institution in Germany can facilitate specific courses on social problems and social institutions within its capabilities [71, 72].

### Strengths and weaknesses

To our knowledge, this is the first qualitative study analysing perceived social problems of patients from the perspective of GPs in combination with the perspective of SPs to explore potentials for a systematic cooperation of the medical and the social care system in Germany. Our participatory, sequential qualitative study design allowed us to examine a maximum of diverse opinions and ideas of 35 participants from various fields.

We assume that there is a selection bias in our study because only GPs who are interested in the topic of social problems and only SPs who are interested in working with GPs participated. Therefore, we tried to maximise the variation of GPs in terms of age, sex, and social status of the local area. In addition, participants differed in their experiences with patients and their collaboration with social care facilities. Regarding SPs, we chose different social facilities worked on the same issue, we selected institution leaders and counsellors, and we looked for cross-institutional stakeholders related to the district. Nevertheless, more information from additional stakeholders may also provide further points of view.

The evolved strategies for a systematic interprofessional collaboration are already a big challenge to implement. In an ideal scenario, all developed steps will be implemented and designated SPs located in the GP practice will act as a link between the GP practice and the social care facilities. Yet for Germany and other countries without good structural conditions, our stepped implementation model could offer a pragmatic approach to using existing resources without requiring substantial changes to the health care system. Any idea for a future, systematic cooperation between GP and SP is primarily burdened with funding issues. However, it is important to note that it would not require many more resources to start with a pragmatic approach. A sustainable and established implementation of improved cooperation between primary care and social care probably requires pooled financing initiatives [73].

Finally, we only considered the patient perspective indirectly. For this reason, we are examining the patient perspective in a separate study, as their behaviour is an essential factor in promoting social care in general practice [74]. Currently, there is a risk that patients expect mainly somatic diagnosis and treatments from their GPs and do not try to address social problems [45] because of stigmatisation [75]. In a further ongoing study, we examine physician assistants' personal perceptions and role identification in treating patients with social problems in primary care.

### Implications

Research is needed to find an appropriate and balanced evidence-based approach to take care of social problems in primary care. To date, there is a lack of evidence across the literature regarding effectiveness [26] as well as mechanisms of interventions to treat social problems [76] because of poor experimental research designs [21]. While it is challenging to quantify specific outcomes for possible changes of individual circumstances and related health benefits [19], there is a plausible causal pathway for social care advice and a strong ethical argument [77]. Tierney et al. [78] note that a theoretical framework for defining serious outcomes is lacking within existing approaches. They suggest that the development of social capital could be an anchor for a framework that could include most intended effects such as developing new knowledge, skills, connections, and empowering patients to engage in activities that make their lives feel satisfying [78]. Wood and colleagues support these ideas and add that social prescribing by link workers may assist people achieve a stronger sense of coherence by strengthening resilience resources [79]. It would be beneficial to combine all these approaches in a logical model to build a comprehensive model of effects for linking SPs and GPs to achieve appropriate outcomes [80]. The goal should be to overcome the eclectic treatment of patients with social problems and to close the gap of professional standards in social care facilities [45].

Furthermore, a specific proactive medical history or screening is essential to detect social problems. Arbitrary approaches lead to divergent estimations between patients and GPs [19]. Corresponding screening instruments have not yet been implemented, but they do exist [81]. Based on a coherent logical model, an established screening of a sophisticated complex intervention around our stepped implementation model for a systematic collaboration between GPs and social workers, we need to develop a high quality randomised controlled trial that shows whether patient-relevant outcomes can be improved in a reliable manner [82].

## Conclusion

Our results suggest that the establishment of interprofessional collaborative structures between GPs and SPs is a promising way to work with patients who have social problems to improve their medical treatment. Based on our stepped implementation model for a systematic cooperation between GPs and SPs, we can recommend first simple steps for implementation in cooperation with civil officials, local politicians, and local representatives of the social and medical care system. These steps should be feasible in most western healthcare systems. Furthermore we need to develop a high quality complex intervention within a randomised controlled trial to create the needed evidence for a wide implementation.

## Data Availability

The datasets created and analysed as part of this study are not publicly available because the transcripts of the qualitative interviews cannot be fully anonymised due to their level of detail. Requests for access to the datasets should be addressed to Thomas Kloppe, t.kloppe@uke.de.

## Abbreviations

FG: Focus group
GP: General practitioner
MP: Mixed-professional
SP: Social professional, social care professional
ICPC: International Classification of Primary Care
ICD: International Statistical Classification of Diseases and Related Health Problems
ICF: International Classification of Functioning, Disability and Health

## References

[1] World Health Organization (WHO). ICF : International classification of functioning, disability and health / World Health Organization. Geneva: World Health Organization (WHO); 2001.

[2] World Health Organization (WHO). International Statistical Classification of Diseases and Related Health Problems (11th Revision). Geneva: World Health Organization (WHO); 2020.

[3] World Organization of National Colleges Academies and Academic Associations of General Practitioners Family Physicians (WONCA). International Classification of Primary Care, 3rd edition (ICPC-3). Brussels: World Organization of National Colleges Academies and Academic Associations of General Practitioners Family Physicians (WONCA); 2020.

[4] Jacobs ZG. Codifying Social Determinants of Health: a Gap in the ICD-10-CM. J Gen Int Med. 2021:3205–7. 10.1007/s11606-021-06742-4. 10.1007/s11606-021-06742-4 PMC8481393 33782895

[5] Marmot M, Bell R (2012). Fair society, healthy lives. Public Health.

[6] Singer S (2018). Psychosocial impact of cancer. Recent Results Cancer Res.

[7] Langbrandtner J, Raspe H, Hüppe A (2016). Chronisch krank und erwerbstätig – Weitere Ergebnisse einer randomisierten kontrollierten Interventionsstudie unter GKV-Versicherten mit chronisch entzündlichen Darmerkrankungen. Z Gastroenterol.

[8] O’Brien R, Wyke S, Watt GGCM, Guthrie B, Mercer SW. The ‘Everyday Work’ of Living with Multimorbidity in Socioeconomically Deprived Areas of Scotland. J Comorb. 2014;4(1):1–10. 10.15256/joc.2014.4.32 PMC5556407 29090148

[9] Zimmermann T, Mews C, Kloppe T, Tetzlaff B, Hadwiger M, von dem Knesebeck O (2018). Social problems in primary health care – prevalence, responses, course of action, and the need for support from a general practitioners’ point of view. Z Evid Fortbild Qual Gesundhwes.

[10] Bikson K, McGuire J, Blue-Howells J, Seldin-Sommer L (2009). Psychosocial problems in primary care: patient and provider perceptions. Soc Work Health Care.

[11] Rattay P, Butschalowsky H, Rommel A, Prütz F, Jordan S, Nowossadeck E, et al. Inanspruchnahme der ambulanten und stationären medizinischen Versorgung in Deutschland. Robert Koch-Institut, Epidemiologie und Gesundheitsberichterstattung. Berlin, Heidelberg: Springer; Bundesgesundheitsbl. 2013:56:832–44. 10.1007/s00103-013-1665-x. 10.1007/s00103-013-1665-x 23703505

[12] Hoebel J, Rattay P, Prütz F, Rommel A, Lampert T (2016). Socioeconomic status and use of outpatient medical care: the case of Germany. PLoS ONE.

[13] Prins MA, Verhaak PFM, Van Der Meer K, Penninx BWJH, Bensing JM. Primary care patients with anxiety and depression: Need for care from the patient’s perspective. J Affect Disord. 2009;119(1–3):163–71. 10.1016/j.jad.2009.03.019 19419771

[14] Boerma WGW, Verhaak PFM (1999). The general practitioner as the first contacted health professional by patients with psychosocial problems: a European study. Psychol Med.

[15] Zantinge EM, Verhaak PF, Bensing JM (2005). The workload of GPs: patients with psychological and somatic problems compared. Fam Pract.

[16] Del Piccolo L, Saltini A, Zimmermann C (1998). Which patients talk about stressful life events and social problems to the general practitioner?. Psychol Med.

[17] Poppleton A, Jones PP, Wright O. Applying social justice, health equity and the social determinants of health to General Practice: A lecture review. InnovAiT: Education and inspiration for general practice. 2022;15(5). 10.1177/1755738020960503.

[18] Popay J, Kowarzik U, Mallinson S, Mackian S, Barker J (2007). Social problems, primary care and pathways to help and support: addressing health inequalities at the individual level. Part II: lay perspectives. J Epidemiol Community Health.

[19] Burrows J, Baxter S, Baird W, Hirst J, Goyder E (2011). Citizens advice in primary care: a qualitative study of the views and experiences of service users and staff. Public Health.

[20] Stumm J, Peter L, Sonntag U, Kumpel L, Heintze C, Dopfmer S. [Non-medical aspects in the care for multimorbid patients in general practice. What kind of support and cooperation is desired? Focus groups with general practitioners in Berlin] Nichtmedizinische Aspekte der Versorgung multimorbider Patient*innen in der Hausarztpraxis. Welche Unterstutzung und Kooperationen werden gewunscht? Fokusgruppen mit Berliner Hausarzt*innen. Z Evid Fortbild Qual Gesundhwes. 2020;158-159:66–73. 10.1016/j.zefq.2020.09.001. 10.1016/j.zefq.2020.09.001 33187897

[21] McGregor J, Mercer SW, Harris FM (2018). Health benefits of primary care social work for adults with complex health and social needs: a systematic review. Health Soc Care Community.

[22] Alderwick H, Dixon J (2019). The NHS long term plan. BMJ.

[23] Workers IAoS. The Role of Social Work in Primary Care. Ireand: Irish Association of Social Workers; 2011.

[24] Network SELHI. Gateway Community Health Centre Counselling 2021 [Available from: https://www.gatewaychc.org/counselling/.

[25] Döbl S, Huggard P, Beddoe L (2015). A hidden jewel: social work in primary health care practice in Aotearoa New Zealand. J Prim Health Care.

[26] Costa A, Sousa CJ, Seabra PRC, Virgolino A, Santos O, Lopes J (2021). Effectiveness of social prescribing programs in the primary health-care context: a systematic literature review. Sustainability.

[27] Golubinski V, Wild E-M, Winter V, Schreyögg J. Once is rarely enough: can social prescribing facilitate adherence to non-clinical community and voluntary sector health services? Empirical evidence from Germany. BMC Public Health. 2020;20(1):1827. 10.1186/s12889-020-09927-4. 10.1186/s12889-020-09927-4 PMC7706247 33256677

[28] Jobst D, Coppola A (2021). Social work and family medicine - an exploratory survey on contacts and cooperation. ZFA - Zeitschrift für Allgemeinmedizin.

[29] Nolting H-D, Ochmann R, Zich K (2021). Gesundheitszentren für Deutschland - Wie ein Neustart in der Primärversorgung gelingen kann.

[30] Golubinski V (2021). Does a doctor’s referral affect individual behavior of using community-based services?. Int J Integr Care.

[31] Jobst D, Fuchs A, Joest A, Nagel N (2007). Anliegen und Wünsche gesunder Patienten - wie gehen Hausärzte damit um?. Das Gesundheitswesen.

[32] Bussche Hvd, Kaduszkiewicz H, Schäfer I, Koller D, Hansen H, Scherer M, et al. Overutilization of ambulatory medical care in the elderly German population? – An empirical study based on national insurance claims data and a review of foreign studies. BMC Health Services Research. 2016;16(1):129. 10.1186/s12913-016-1357-y. 10.1186/s12913-016-1357-y PMC4831189 27074709

[33] Reeves S, Xyrichis A, Zwarenstein M (2018). Teamwork, collaboration, coordination, and networking: why we need to distinguish between different types of interprofessional practice. J Interprof Care.

[34] World Health Organization (WHO). Framework for action on interprofessional education & collaborative practice. In: Health DoHRf, editor. Geneva: World Health Organization (WHO); 2010.

[35] Pohontsch NJ, Muller V, Brandner S, Karlheim C, Junger S, Klindtworth K (2018). Group discussions in health services research - part 1: introduction and deliberations on selection of method and planning. Gesundheitswesen.

[36] Onwuegbuzie AJ, Dickinson WB, Leech NL, Zoran AG (2009). A qualitative framework for collecting and analyzing data in focus group research. Int J Qual Methods.

[37] Marshall MN. Sampling for qualitative research. Fam Pract. 1996;13(6):522–5. 10.1093/fampra/13.6.522. 10.1093/fampra/13.6.522 9023528

[38] Lüde JV, Kaiser A (2020). Sozialmonitoring Integrierte Stadtteilentwicklung Bericht 2020.

[39] Kuckartz U (2014). Qualitative Text Analysis: A Guide to Methods, Practice & Using Software.

[40] Software V (2020). MAXQDA 2020.

[41] Ommen O, Thuem S, Pfaff H, Janssen C (2011). The relationship between social support, shared decision-making and patient’s trust in doctors: a cross-sectional survey of 2,197 inpatients using the Cologne Patient Questionnaire. Int J Public Health.

[42] Jobst D, Joos S. Soziale Patientenanliegen – eine Erhebung in Hausarztpraxen. Zeitschrift für Allgemeinmedizin. 2014;90(12):496–501. 10.3238/zfa.2014.0496-0501.

[43] Wilfer T, Braungardt T, Schneider W. Soziale Probleme in der hausärztlichen Praxis. Göttingen: Zeitschrift für Psychosomatische Medizin und Psychotherapie; 2018. p. 250–61. https://www.jstor.org/stable/26541258. 10.13109/zptm.2018.64.3.250 30829155

[44] Warth J, Puth M-T, Zier U, Beckmann N, Porz J, Tillmann J, et al. Patient-physician communication about financial problems: A cross-sectional study among over-indebted individuals. PLoS ONE. 2020;15(5):e0232716. 10.1371/journal.pone.0232716 PMC7199951 32369528

[45] Islam MM. Social Prescribing—An Effort to Apply a Common Knowledge: Impelling Forces and Challenges. Front Public Health. 2020;8:515469. 10.3389/fpubh.2020.515469. 10.3389/fpubh.2020.515469 PMC7728793 33330299

[46] McCall-Hosenfeld JS, Weisman CS, Perry AN, Hillemeier MM, Chuang CH. “I Just Keep My Antennae Out”: how rural primary care physicians respond to intimate partner violence. J Interpers Violence. 2014;29(14):2670–94. 10.1177/0886260513517299 PMC4121375 24424251

[47] Jovicic A, McPherson S (2020). To support and not to cure: general practitioner management of loneliness. Health Soc Care Community.

[48] Miller R, Weir C, Gulati S (2018). Transforming primary care: scoping review of research and practice. J Integr Care.

[49] Mercer SW, Fitzpatrick B, Grant L, Chng NR, McConnachie A, Bakhshi A (2019). Effectiveness of community-links practitioners in areas of high socioeconomic deprivation. Ann Fam Med.

[50] Nottingham C, Dougall R (2007). A close and practical association with the medical profession: Scottish medical social workers and social medicine, 1940–1975. Med Hist.

[51] Ratoff L, Pearson B (1970). Social case-work in general practice: an alternative approach. BMJ.

[52] Seaton J, Jones A, Johnston C, Francis K. Allied health professionals' perceptions of interprofessional collaboration in primary health care: an integrative review. J Interprof Care. 2020;35(2):1–12. 10.1080/13561820.2020.1732311. 10.1080/13561820.2020.1732311 32297811

[53] Abbott S, Hobby L, Cotter S (2006). What is the impact on individual health of services in general practice settings which offer welfare benefits advice?. Health Soc Care Community.

[54] Wagle K, Cottingham AH, Butler D, Grover J, Litzelman DK. Understanding social workers’ hidden roles in medication safety for older adults: A qualitative study. Soc Work Health Care. 2021;378:1–18. 10.1016/S0140-6736(11)61179-3. 10.1080/00981389.2021.1900023 33730520

[55] Feder G, Davies RA, Baird K, Dunne D, Eldridge S, Griffiths C (2011). Identification and Referral to Improve Safety (IRIS) of women experiencing domestic violence with a primary care training and support programme: a cluster randomised controlled trial. The Lancet.

[56] Health care resources. 2016. Available from: https://www.oecd-ilibrary.org/content/data/data-00541-en.

[57] Mangan C, Miller R, Cooper J (2014). Time for some home truths – exploring the relationship between GPs and social workers. J Integr Care.

[58] Payne RA, Mendonca SC, Elliott MN, Saunders CL, Edwards DA, Marshall M (2020). Development and validation of the Cambridge Multimorbidity Score. CMAJ.

[59] Bickerdike L, Booth A, Wilson PM, Farley K, Wright K (2017). Social prescribing: less rhetoric and more reality. A systematic review of the evidence. BMJ Open.

[60] Hamilton-West K, Gadsby E, Zaremba N, Jaswal S (2019). Evaluability assessments as an approach to examining social prescribing. Health Soc Care Community.

[61] Naqvi D, Malik A, Al-Zubaidy M, Naqvi F, Tahir A, Tarfiee A (2019). The general practice perspective on barriers to integration between primary and social care: a London, United Kingdom-based qualitative interview study. BMJ Open.

[62] Frostick C, Bertotti M. The frontline of social prescribing - How do we ensure Link Workers can work safely and effectively within primary care?. Chronic Illn. 2019;17(4). 10.1177/1742395319882068. 10.1177/1742395319882068 31623451

[63] Ashcroft R, McMillan C, Ambrose-Miller W, McKee R, Brown JB (2018). The emerging role of social work in primary health care: a survey of social workers in Ontario family health teams. Health Soc Work.

[64] Byhoff E, Garg A, Pellicer M, Diaz Y, Yoon GH, Charns MP, et al. Provider and staff feedback on screening for social and behavioral determinants of health for pediatric patients. J Am Board Fam Med. 2019;32(3):297–306. 10.3122/jabfm.2019.03.180276. 10.3122/jabfm.2019.03.180276 PMC12794923 31068394

[65] Cheetham M, Van der Graaf P, Khazaeli B, Gibson E, Wiseman A, Rushmer R. “It was the whole picture” a mixed methods study of successful components in an integrated wellness service in North East England. BMC Health Serv Res. 2018;18(1):200. 10.1186/s12913-018-3007-z PMC5863899 29566687

[66] Grant C (2000). A randomised controlled trial and economic evaluation of a referrals facilitator between primary care and the voluntary sector. BMJ.

[67] Comas-Herrera A, Fernandez J-L, Hancock R, Hatton C, Knapp M, Mcdaid D (2020). COVID-19: implications for the support of people with social care needs in England. J Aging Soc Policy.

[68] Schultz R, Brostrøm Kousgaard M, Davidsen AS. “We have two different agendas”: the views of general practitioners, social workers and hospital staff on interprofessional coordination for patients with chronic widespread pain. Journal of Interprofessional Care. 2020;35(2):1–9. 10.1080/13561820.2020.1749576. 10.1080/13561820.2020.1749576 32297802

[69] O’Carroll V, McSwiggan L, Campbell M. Health and social care professionals’ attitudes to interprofessional working and interprofessional education: a literature review. J Interprof Care. 2016;30(1):42–9. 10.3109/13561820.2015.1051614 26709753

[70] Hommes F, Drees S, Geffert K, Philipsborn Pv, Stratil J. Representation of Social Determinants of Health in German Medical Education. Gesundheitswesen: Georg Thieme Verlag KG Stuttgart; 2019. 10.1055/a-1005-7082.

[71] Koch-Gromus U, Guse AH, Van Den Bussche H (2018). Medizinische Ausbildung in Bewegung. Bundesgesundheitsblatt - Gesundheitsforschung - Gesundheitsschutz.

[72] Kloppe T, Zimmermann T, Mews C, Tetzlaff B, Scherer M (2021). Krank, arm, einsam und arbeitslos – Verbindung von hausärztlicher Praxis und sozialem Hilfesystem – ein Konzept für Aus- und Fortbildung. Gesundheitswesen.

[73] Sandhu S, Alderwick H, Gottlieb LM. Financing Approaches to Social Prescribing Programs in England and the United States. New York: The Milbank Quarterly; 2022. 10.1111/1468-0009.12562. 10.1111/1468-0009.12562 PMC9205663 35348249

[74] Kriegel J, Rissbacher C, Pölzl A, Tuttle-Weidinger L, Reckwitz N (2020). Levers for integrating social work into primary healthcare networks in Austria. Health Policy.

[75] Pescheny J, Randhawa G, Pappas Y. Patient uptake and adherence to social prescribing: a qualitative study. BJGP Open. 2018;2(3):bjgpopen18X101598. 10.3399/bjgpopen18X101598 PMC6189784 30564731

[76] Chatterjee HJ, Camic PM, Lockyer B, Thomson LJM (2017). Non-clinical community interventions: a systematised review of social prescribing schemes. Arts Health.

[77] Abbott S (2002). Prescribing welfare benefits advice in primary care: is it a health intervention, and if so, what sort?. J Public Health.

[78] Tierney S, Wong G, Roberts N, Boylan AM, Park S, Abrams R, et al. Supporting social prescribing in primary care by linking people to local assets: A realist review. BMC Medicine: BioMed Central Ltd. 2020;18:49. 10.1186/s12916-020-1510-7. 10.1186/s12916-020-1510-7 PMC7068902 32164681

[79] Wood E, Ohlsen S, Fenton S-J, Connell J, Weich S. Social prescribing for people with complex needs: a realist evaluation. BMC Fam Pract. 2021;22(1):53. 10.1186/s12875-021-01407-x. 10.1186/s12875-021-01407-x PMC7977569 33736591

[80] Bertotti M, Frostick C, Hutt P, Sohanpal R, Carnes D. A realist evaluation of social prescribing: an exploration into the context and mechanisms underpinning a pathway linking primary care with the voluntary sector. Primary Health Care Res Devel. 2017;19:232–45. 10.1017/S1463423617000706. 10.1017/S1463423617000706 PMC5904290 29215328

[81] Schwenker R, Kroeber ES, Deutsch T, Frese T, Unverzagt S (2021). Identifying patients with psychosocial problems in general practice: a scoping review protocol. BMJ Open.

[82] Goodwin N. Understanding and Evaluating the Implementation of Integrated Care: A ‘Three Pipe’ Problem. Int J Integ Care. 2016;16(4):19. 10.5334/ijic.2609. PMC5354212 28316558

